# Lemmel Syndrome Presenting with Transient Cholangitis and Bacteremia: A Case Report

**DOI:** 10.7759/cureus.93782

**Published:** 2025-10-03

**Authors:** Feras Buhusayen, Aysha Hassan Ali, Amreen Mustafa, Hend Almahmood, Thamer Alabbasi

**Affiliations:** 1 General Surgery, Bahrain Defence Force Hospital, Royal Medical Services, Riffa, BHR; 2 Respiratory Medicine, Sandwell and West Birmingham NHS Trust, Birmingham, GBR; 3 Medicine, Bahrain Defence Force Hospital, Royal Medical Services, Riffa, BHR

**Keywords:** cholangitis, duodenal diverticulum, endoscopic retrograde cholangiopancreatography, lemmel syndrome, obstructive jaundice, periampullary duodenal diverticulum

## Abstract

Lemmel syndrome is a rare disease characterized by mechanical obstruction of the bile duct secondary to a periampullary duodenal diverticulum. We present the case of a 74-year-old male patient admitted with generalized abdominal pain and fever. His initial blood work revealed derangement of liver function tests (LFTs) and leukocytosis. Imaging, including ultrasound and contrast-enhanced computed tomography (CECT) of the abdomen and pelvis, showed multiple gallstones without biliary dilation and ruled out any hepatobiliary obstruction and micro-abscesses. However, an upper gastrointestinal endoscopy identified a duodenal diverticulum. Given the patient’s bacteremia and the presence of duodenal diverticula without any evidence of an obstructive cause, Lemmel syndrome with transient cholangitis was considered the most likely diagnosis. Unlike most published reports of Lemmel syndrome presenting with persistent obstructive jaundice, our case is distinctive in its transient presentation complicated by bacteremia, which posed diagnostic challenges and required multimodal imaging for confirmation.

## Introduction

Periampullary diverticula are extraluminal outpouches in the mucosa of the duodenum surrounding the ampulla of Vater [1]. Most cases of periampullary diverticuli are asymptomatic, but complications can arise and present as cholangitis or pancreatitis [2,3]. Diagnosis of periampullary diverticuli is often incidental during endoscopy or imaging studies for abdominal pain [3]. When a patient with periampullary diverticuli is symptomatic, imaging modalities such as computed tomography (CT) scans and magnetic resonance imaging (MRI) can help make the diagnosis, but a side-viewing endoscope provides direct visualization of the diverticuli and also allows for the potential treatment of the disease [4]. Lemmel syndrome is a complication of periampullary diverticuli where the diverticuli cause mechanical obstruction of the pancreatic and common bile ducts, leading to obstructive jaundice without evidence of choledocholithiasis or malignant biliary obstruction [5]. These patients are at risk for further complications such as sepsis and perforation [6]. Early diagnosis and treatment of these patients can help prevent further complications. We are presenting a case of Lemmel syndrome with obstructive jaundice that was complicated by cholangitis and bacteremia.

## Case presentation

A 74-year-old male patient with a past medical history of hypertension, type 2 diabetes mellitus, and dyslipidemia presented with a one-day history of generalized abdominal pain, predominantly in the left lower quadrant, associated with fever and chills. He also reported a single episode of loose stools but denied weight loss, changes in appetite, nausea, vomiting, or dysphagia. His medication history included oral antidiabetic medications and vitamin D and B12 supplements. He was a lifelong non-smoker but reported consuming one to two units of alcohol daily for the past 50 years. The patient had returned from Egypt a couple of days ago. There was no recent history of contact with sick individuals. On examination, he was hemodynamically stable; vital signs were as follows: blood pressure of 130/85 mmHg, heart rate of 88 beats per minute, temperature of 37°C, respiratory rate of 18 breaths per minute, and SpO2 of 99% on room air. He had a tinge of scleral icterus. His abdomen was soft and nontender, and no masses were felt. Otherwise, the physical exam was unremarkable. Laboratory investigations revealed leukocytosis (WBC: 22 x 10⁹/L), hyperbilirubinemia (total bilirubin: 74 µmol/L, direct bilirubin: 55 µmol/L), and elevated liver enzymes (alkaline phosphatase (ALP): 128 U/L; aspartate aminotransferase (AST): 344 U/L; alanine aminotransferase (ALT): 344 U/L; gamma-glutamyl transferase (GGT): 720 U/L).

**Figure 1:**
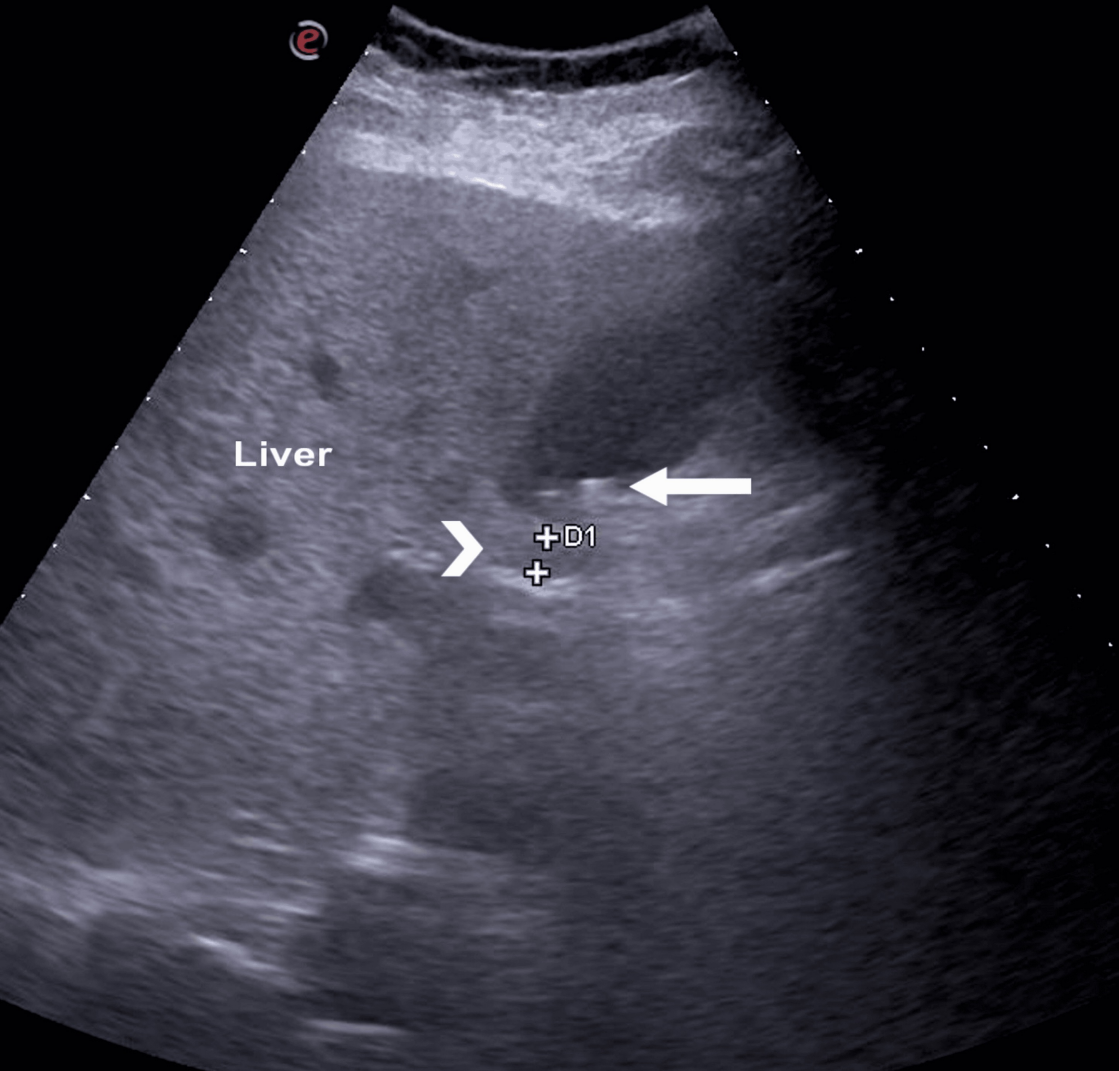
Ultrasound image showing the liver, multiple sub-centimeter gallstones (solid arrow), and the common bile duct (arrowhead).

**Figure 2:**
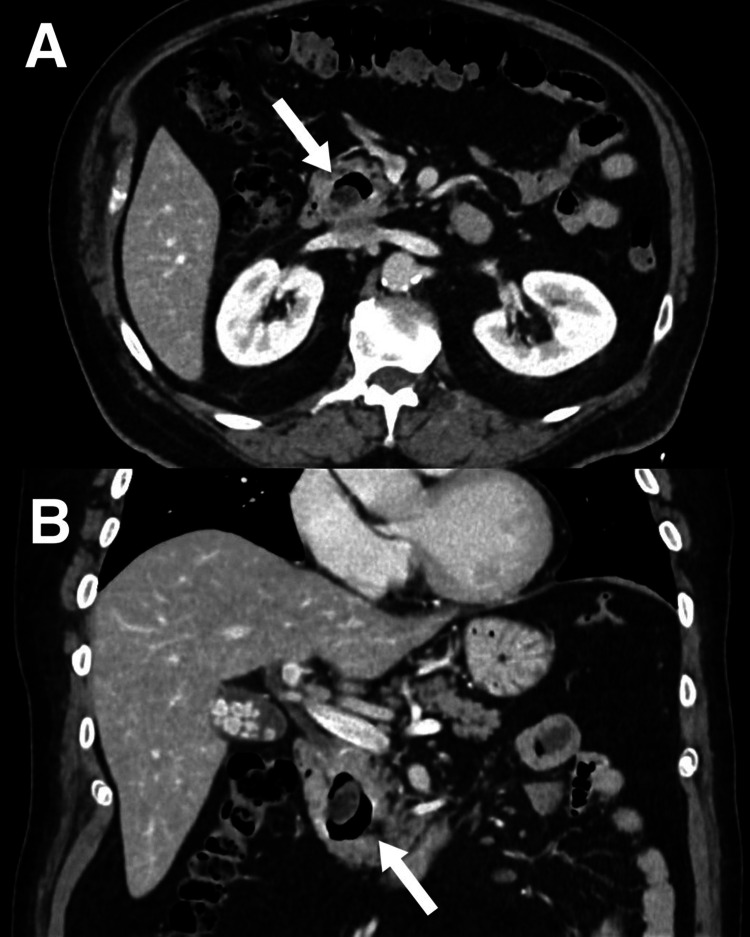
Axial (A) and coronal (B) IV contrast-enhanced CT of the abdomen showing a duodenal diverticulum, noted adjacent to the distal end of the common bile duct. The diverticulum measures 2.2 x 1.5 cm.

**Figure 3:**
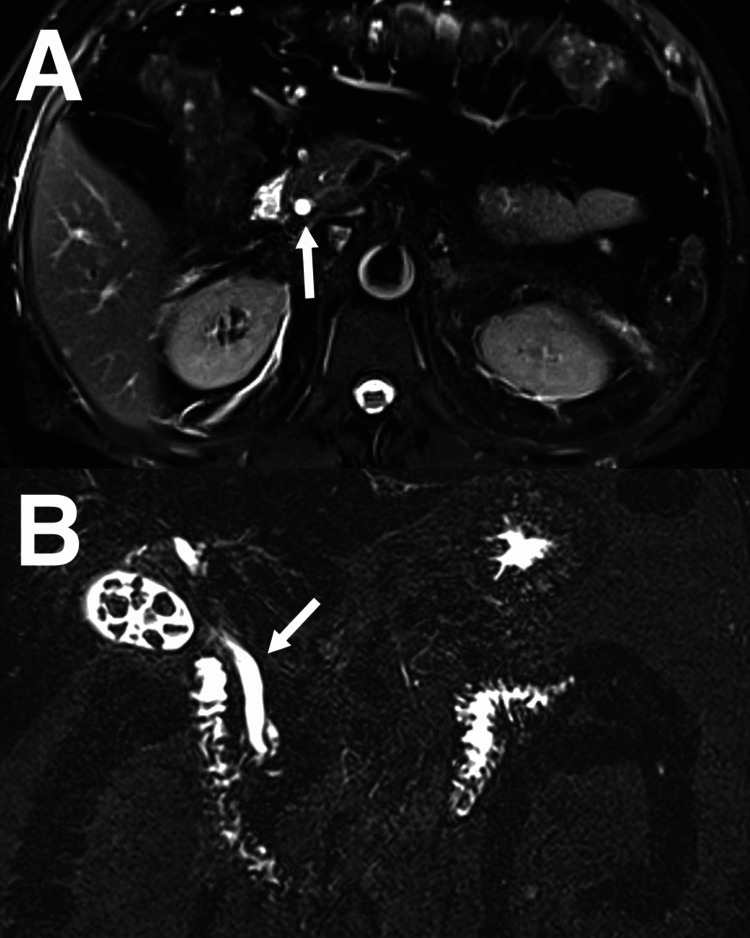
Axial (A) and coronal (B) magnetic resonance cholangiopancreatography studies showing a prominent common bile duct (CBD), measuring 7 mm. In addition, the distal end of the CBD appeared irregular. These findings are attributed to the extrinsic compression of the CBD by the duodenal diverticulum. No filling defect or stone seen in the proximal CBD or common hepatic duct. No mass lesions or dilatation of the pancreatic duct or intrahepatic bile ducts are seen.

Abdominal ultrasound showed multiple sub-centimeter gallstones with normal gallbladder wall thickness and no fluid collection, no signs of ductal dilatation, and the common bile duct (CBD) measuring 6.3 mm (Figure 1). Blood culture, hepatitis profile, coagulation profile, Epstein-Barr virus (EBV), and cytomegalovirus (CMV) serology were drawn. A triphasic liver CT confirmed the absence of liver micro-abscesses and bile duct strictures. Contrast-enhanced CT (CECT) of the abdomen and pelvis (Figure 2) showed a large, thin-walled, ovoid outpouching arising from the medial wall of the second portion of the duodenum, measuring 2.2 x 1.5 cm, containing air, and consistent with a duodenal diverticulum. It is noted adjacent to the distal end of the CBD. Magnetic resonance cholangiopancreatography (MRCP) (Figure 3) was performed due to diagnostic uncertainty and suspected obstructive cholestasis, which confirmed the presence of a duodenal diverticulum located near the ampulla of Vater. A prominent CBD measuring 7 mm was also noted. In addition, the distal end of the CBD appeared irregular. These findings are attributed to the extrinsic compression of the CBD by the duodenal diverticulum. No filling defect or stone seen in the proximal CBD or common hepatic duct (CHD). No mass lesions were seen. No dilatation of the pancreatic duct or intrahepatic bile ducts was noted.

Empirical intravenous piperacillin/tazobactam was initiated. Blood cultures subsequently grew *Escherichia coli*, confirming bacteremia. Hepatitis A, B, and C serologies were negative, with EBV and CMV pending. Serial blood cultures were negative following the initiation of antibiotics. The patient had persistent low-grade fever, leukocytosis, and abnormal liver function tests (LFTs). Considering his history of duodenal diverticulum and a lack of another clear cause, Lemmel syndrome with transient cholangitis and bacteremia was considered the most likely diagnosis.

Final laboratory investigations prior to discharge demonstrated a steady improvement in inflammatory markers and liver function tests. The white cell counts normalized, declining from 18.78×10⁹/L to 7.1×10⁹/L. Liver transaminases, AST and ALT, were 122 U/L and 131 U/L, respectively, marking a decline from their peak values of 344 U/L and 214 U/L. Similarly, total bilirubin decreased from 59 µmol/L to 17.4 µmol/L, while GGT improved from 720 U/L to 552 U/L, reflecting overall biochemical recovery. Table 1 demonstrates the laboratory findings. 

**Table 1:** Summary of all the laboratory values WBC: white blood cell count; ALP: alkaline phosphatase; AST: aspartate aminotransferase; ALT: alanine aminotransferase; GGT: gamma-glutamyl transferase

Laboratory Test	Initial Value	Intermediate Peak/Follow-up	Discharge Value	Normal Range
WBC (×10⁹/L)	22	18.78	7.1	4.0 – 11.0
Total Bilirubin (µmol/L)	74	59	17.4	3 – 20
Direct Bilirubin (µmol/L)	55	—	—	0 – 5
ALP (U/L)	128	—	—	44 – 147
AST (U/L)	344	—	122	0 – 40
ALT (U/L)	344	214	131	0 – 40
GGT (U/L)	720	—	552	9 – 48

**Figure 4:**
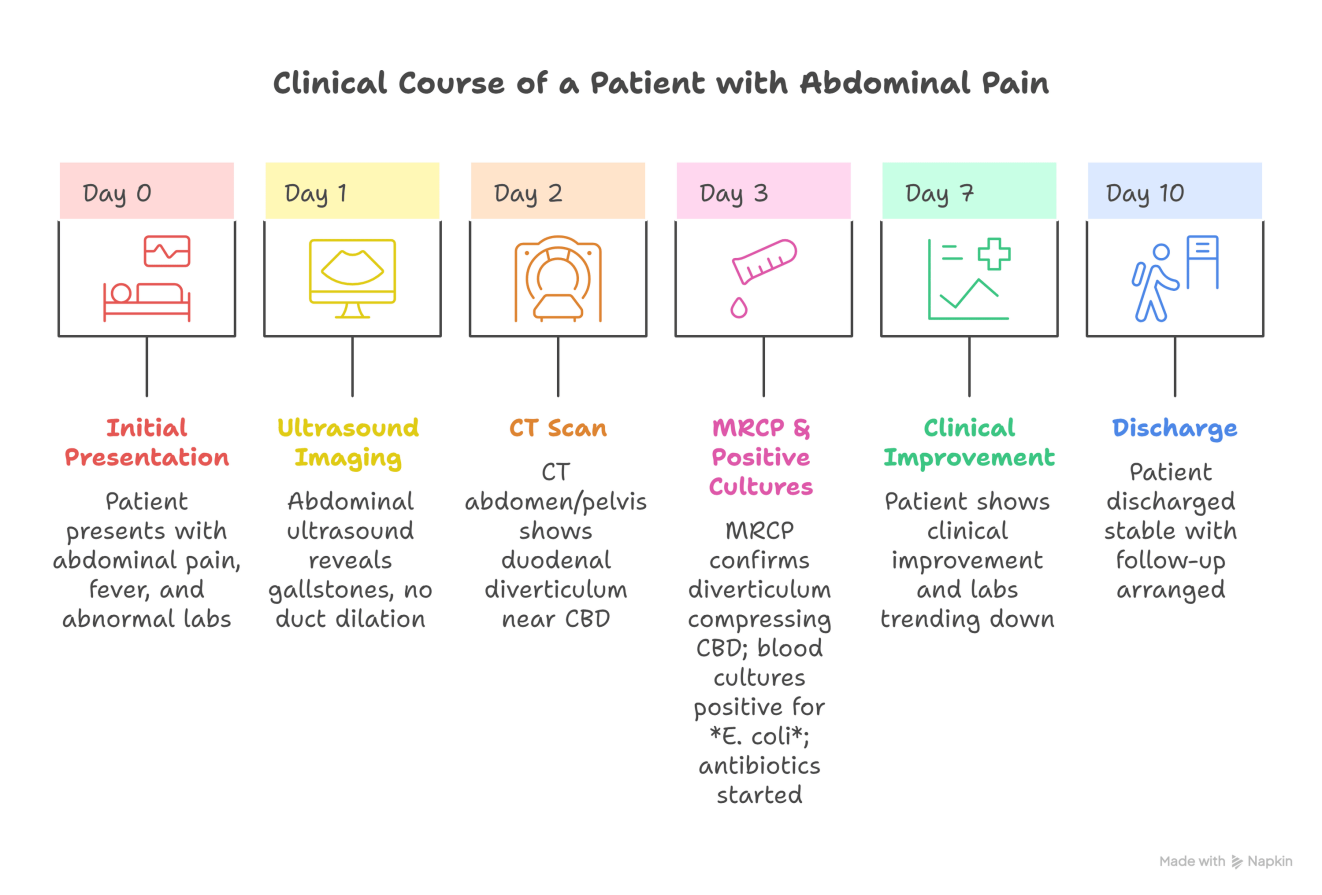
Summary of the patient’s clinical course, including presentation, diagnostic workup, management, and outcome CBD: common bile duct; MRCP: magnetic resonance cholangiopancreatography; *E. coli*: *Escherichia coli*

Following his clinical improvement with normalization of white cell count and declining bilirubin levels, the patient was discharged in a stable condition, and an infectious disease consultation was requested to determine the optimal duration of antibiotic therapy. A summary of the patient’s clinical course, including presentation, diagnostic workup, management, and outcome, is provided in Figure 4.

## Discussion

Diverticula of the gastrointestinal tract are protrusions or sac-like herniations of the intestinal wall that can develop at various locations, with the colon being the most commonly affected site, followed by the duodenum [2]. Duodenal diverticula are categorized according to their anatomical position, with periampullary diverticula, located near the ampulla of Vater, being the most frequently encountered type [2,3]. While these periampullary diverticula are typically silent and discovered incidentally, they can occasionally lead to complications [7]. These complications are either pancreaticobiliary or non-pancreaticobiliary in nature [7]. The latter are relatively uncommon and may involve inflammation (diverticulitis), bleeding, perforation, or the formation of fistulas [7]. Pancreaticobiliary complications, on the other hand, may manifest as recurrent cholelithiasis or choledocholithiasis, obstructive jaundice, ascending cholangitis, or acute pancreatitis [7].

Lemmel syndrome, obstruction of the CBD by a periampullary duodenal diverticulum, is a rare cause of cholangitis and obstructive jaundice [8]. Its infrequent occurrence and non-specific presentation often mimic more common causes (e.g., choledocholithiasis or malignancy) [9-11]. In this 74-year-old man, the intermittent nature of biliary obstruction led to transient cholangitis episodes with bacteremia, making the diagnosis challenging. Clinicians initially faced an obstructive jaundice picture with elevated cholestatic liver enzymes and infection markers, but no obvious stone, tumor, or stricture on initial imaging, a scenario that demands high suspicion for an atypical obstructing lesion such as a duodenal diverticulum.

Published cases of Lemmel syndrome predominantly involve older adults, typically in the sixth to eighth decade of life [6,12,13]​. This aligns with our 74-year-old patient. There is a notable variation in clinical presentation. Some patients present with insidious, intermittent abdominal pain and mild jaundice over months [8,1,13], whereas others, like this case, present acutely with Charcot’s triad or even sepsis from ascending cholangitis [12, [6]. A literature review found jaundice in about 68% to 100% of reported cases and fever (cholangitis) in roughly 50% [4,14]. Abdominal pain is common but not universal, and a subset of patients have only subtle symptoms or normal exams despite significant biliary obstruction [14]. This spectrum suggests Lemmel syndrome can range from an incidental finding with mild symptoms to life-threatening cholangitis, mirroring the transient yet severe episodes observed in our patient.

The diagnosis across reported cases has been made using various modalities, often requiring more than one. Transabdominal ultrasound frequently shows biliary dilatation without an obvious cause, as in our case, but it may miss duodenal diverticula due to bowel gas or a lack of a distinct mass lesion. CT is a common initial tool (one review noted CT was the first diagnostic modality in ~47% of cases) and can demonstrate a periampullary diverticulum as an air- or contrast-filled outpouching compressing the bile duct [4]. CT findings typically include a dilated CBD with an adjacent duodenal diverticulum [4]. In one series, all patients had a dilated CBD (mean ~12.5 mm) visible on CT scan [4]. MRCP provides excellent anatomic detail and helps exclude choledocholithiasis or neoplasm; for example, MRCP in published cases often confirmed the diverticulum causing a “double-duct sign” (simultaneous biliary and pancreatic duct dilation) with no internal filling defects [8]. Notably, MRCP is considered a non-invasive gold standard by many for delineating the biliary tree and periampullary region in Lemmel syndrome. Finally, endoscopic evaluation is frequently decisive: side-viewing endoscopy (endoscopic retrograde cholangiopancreatography (ERCP) or duodenoscopy) can directly identify the periampullary diverticulum and the papilla, sometimes found within or at the rim of the diverticulum [12]. In fact, ERCP is often described as the diagnostic gold standard, as it allows direct visualization and the option to perform therapy in the same session [8]. Many case reports document that ERCP cholangiography reveals a dilated biliary system without stones, confirming extrinsic compression​ [8]. In summary, a combination of imaging modalities is usually employed, often ultrasound or CT to raise suspicion, MRCP to characterize, and ERCP to confirm and treat, each case requiring a tailored approach.

Despite variations in presentation, outcomes in documented cases have been favorable when Lemmel syndrome is correctly identified and managed. Endoscopic intervention is the mainstay in most reports. Therapeutic ERCP (with sphincterotomy and/or stent placement) reliably resolves the biliary obstruction and treats acute cholangitis, with one study citing a cannulation success rate of ~95% even when the papilla is within a diverticulum [15]. Following endoscopic therapy, recurrent cholangitis is uncommon; the literature indicates that once adequate biliary drainage is established, patients usually remain symptom-free on follow-up [15]​. In contrast, cases that were initially misdiagnosed or untreated suffered repetitive bouts of jaundice/cholangitis until the diverticulum was recognized [10]​. Surgical management (such as diverticulectomy or biliary bypass) has been reported in a minority of cases, typically those with refractory or complicated disease (e.g., a diverticulum that is inflamed or causing repeated obstruction despite endoscopy) [6,16]. However, most authors agree that surgery is not first-line: asymptomatic or incidentally found periampullary diverticula should be left alone, and even symptomatic Lemmel syndrome often resolves with less invasive measures. For example, one review emphasized that Lemmel syndrome can most often be managed conservatively if caught early (as in our case), except in the setting of emergent complications [16]. Comparing across cases, the consistent lesson is that timely endoscopic therapy leads to excellent outcomes, whereas unnecessary surgical exploration can be avoided by recognizing this rare syndrome.

## Conclusions

Lemmel syndrome, though rare, should be considered in the differential diagnosis of unexplained obstructive jaundice, especially in elderly patients when common causes such as stones or malignancy have been excluded. The presence of a periampullary duodenal diverticulum may not be evident on initial imaging, and biliary dilation may be subtle or absent, leading to potential diagnostic delays. Clinicians should maintain a high index of suspicion and pursue advanced imaging like CT or MRCP, which can better visualize diverticula and their effect on the biliary tree. Endoscopic evaluation remains crucial both for confirming the diagnosis and providing effective treatment through biliary drainage. Increased awareness of this condition can prevent unnecessary surgical exploration and ensure timely, minimally invasive management.
